# From personal crisis care to convenience shopping: an interpretive description of the experiences of people with mental illness and addictions in community pharmacies

**DOI:** 10.1186/s12913-016-1817-4

**Published:** 2016-10-12

**Authors:** Andrea L. Murphy, Ruth Martin-Misener, Stan P. Kutcher, Claire L. O’Reilly, Timothy F. Chen, David M. Gardner

**Affiliations:** 1 College of Pharmacy and Department of Psychiatry, Dalhousie University, 5968 College St., PO Box 15000, Halifax, NS B3H 4R2 Canada; 2 School of Nursing, Dalhousie University, 5869 University Ave., PO Box 15000, Halifax, NS B3H 4R2 Canada; 3 Sun Life Financial Chair in Adolescent Mental Health, Dalhousie University/IWK Health Centre, 5850 University Ave., PO Box 9700, Halifax, NS B3K 6R8 Canada; 4 Faculty of Pharmacy, University of Sydney, Pharmacy and Bank Building (A15), Camperdown Campus, The University of Sydney, Sydney, NSW 2006 Australia; 5 Department of Psychiatry and College of Pharmacy, Dalhousie University, QEII HSC, AJLB 7517, 5909 Veterans’ Memorial Lane, Halifax, NS B3H 2E2 Canada

**Keywords:** Mental disorders, Pharmacy, Qualitative research, Patient preference, Decision making, Suicide

## Abstract

**Background:**

The role of community pharmacists is changing globally with pharmacists engaging in more clinically-oriented roles, including in mental health care. Pharmacists’ interventions have been shown to improve mental health related outcomes but various barriers can limit pharmacists in their care of patients. We aimed to explore the experiences of people with lived experience of mental illness and addictions in community pharmacies to generate findings to inform practice improvements.

**Methods:**

We used interpretive description methodology with analytic procedures of thematic analysis to explore the experiences of people with lived experience of mental illness and addictions with community pharmacy services. Participants were recruited through multiple mechanisms (e.g., paper and online advertisements), offered honorarium for their time, and given the option of a focus group or interview for participation in our study. Data were gathered during July to September of 2012. Interviews and focus groups were audio-recorded, transcribed verbatim, and analyzed by two researchers.

**Results:**

We collected approximately nine hours of audio data from 18 individuals in two focus groups (*n* = 12) and six individual interviews. Fourteen participants were female and the average age was 41 years (range 24 to 57 years). Expectations, decision-making, and supports were identified as central themes underlying the community pharmacy experiences of people with lived experience of mental illness and addictions. Eight subthemes were identified including: relationships with pharmacy staff; patient’s role in the pharmacist-patient relationship; crisis and triage; privacy and confidentiality; time; stigma and judgment; medication-related and other services; and transparency.

**Conclusions:**

People with lived experience of mental illness and addictions demonstrate a high regard and respect for pharmacist’s knowledge and abilities but hold conservative expectations of pharmacy health services shaped by experience, observations, and assumptions. To some extent, expectation management occurs with the recognition of the demands on pharmacists and constraints inherent to community pharmacy practice. Relationships with pharmacy staff are critical to people with lived experience and influence their decision-making. Research in the area of pharmacists’ roles in crises and triage, especially in the area of suicide assessment and mitigation, is needed urgently.

**Electronic supplementary material:**

The online version of this article (doi:10.1186/s12913-016-1817-4) contains supplementary material, which is available to authorized users.

## Background

Pharmacists have been identified as playing important roles in primary mental health care as part of the primary mental health care team [1]. The enhanced roles for pharmacists in mental health care are increasingly clinically-oriented, collaborative roles, [2] in keeping with current standards of practice, and offer opportunities to improve gaps in the mental health system related to effectiveness, efficiency, and equality of care [3, 4]. Professional role revision in the health system can also help to meet public demands and changing expectations [5]. Public expectations of pharmacists, who are trusted, accessible, and in frequent contact with the public [6–8] have been evolving with recognition that pharmacists have roles beyond the “drug expert” [9]. Understanding the public and patients’ perspectives on pharmacy services is important, especially with changes in roles, in order to help inform how and under what circumstances pharmacists can efficiently and effectively collaborate in providing services to people with lived experience of mental illness and addictions. Although existing research and policy documents indicate positive impacts of pharmacists’ roles in mental health care [1, 10], there are also findings of significant barriers (e.g., privacy, stigma, limited staffing) that restrict pharmacists in their care of patients with mental illness and addictions [11–21].

There is limited knowledge on how these various challenges and opportunities in pharmacy practice impact patients with mental illness and addictions in their experiences of pharmacy services. Surveys [13, 14, 22, 23] have been conducted and primarily focused on descriptive statistics regarding the nature and range of pharmacy services offered, and other constructs including stigma and self-reported satisfaction. Few qualitative studies regarding patients’ experiences with pharmacy services have been conducted [19, 24]. For example, Knox et al. conducted computer-assisted telephone interviews with 210 Australian people regarding their experiences, expectations, and satisfaction with technical and functional quality of community pharmacy services for people living with and/or caring for someone with lived experience of mental illness [19]. Technical quality was assessed using self-reported perceptions of wait times, receipt of verbal and written advice, and if received, the characteristics of the content. Functional quality was assessed based on self-reported satisfaction with interactions and perceptions of services including what was desirable and areas for improvement. Data were gathered based on an instrument with 48 rating scales, 38 multiple-choice checklists, and 19 open-ended questions, which included thematic analysis through a framework for patient-centred care. Participants desired efficiency, consistent, and personalized pharmacy services. Issues with technical service delivery and experiences of stigma were reported as undesirable and an area requiring improvement [19]. Treloar et al. [24] reported findings of a thematic content analysis of interviews with 25 methadone clients in Australia, of whom 21 received takeaway doses from pharmacies. Participants indicated that trust in the relationship with pharmacists was important with takeaway dosing and helped to provide encouragement for patients. Accessing daily methadone doses presented both conveniences and inconveniences for many participants. Studies such as these demonstrate that a host of factors (e.g., inefficiencies, information quality, privacy and confidentiality, and stigma) can influence experiences of people with mental illness and addictions in community pharmacy settings. Literature is also available describing patients’ perspectives and expectations of pharmacy services generally [25], within the umbrella of chronic conditions [26, 27], or more recent pharmacist-delivered public health initiatives [9] (e.g., smoking cessation [28], alcohol screening [29–31]). These studies contribute additional knowledge regarding the patient experience in general, including information on preferences for services, relative importance of services offered, and willingness to engage in services, but more research specifically examining the perspectives of people with mental illness and addictions in pharmacy contexts is needed to inform practice and systems’ improvements. This is especially important in this group of people given established issues with accessibility of care, inequalities in health care service delivery, and poorer health outcomes affecting this population [32–41].

To contribute to the knowledge in this area, we conducted a qualitative study to explore the experiences of people with lived experience of mental illness and addictions, or their support people or caregivers, with community pharmacy services. The goals of conducting this study were twofold: to explore and understand the experiences of people with lived experience of mental illness and addictions when receiving community pharmacy services; and to generate new findings that can inform interventions for practice that are underpinned by a behaviour framework [42] to help pharmacists improve their care of people with lived experience of mental illness and addictions.

## Methods

### Design

We used interpretive description methodology [43], with analytic procedures of thematic analysis [44, 45] to explore the experiences of people with lived experience of mental illness and addictions with community pharmacy services. Interpretive description methodology was appropriate for our goals for reasons including our acknowledgement of the socially constructed element of human experience and that these experiences are part of multiple constructed realities [43]. This methodology is in keeping with the theory and models of behaviour that influenced our work through the research process including Role Theory [46], the Theoretical Domains Framework [47], and the Behaviour Change Wheel [42]. Our intention in studying this phenomenon was also pragmatic with the goal of using the findings to inform future intervention development for practice-based improvements in mental health care, which is in keeping with interpretive description [43].

### Study participants

We used convenience sampling and recruited people with lived experience of mental illness and addictions, and their caregivers, herein abbreviated and referred to as PLEs for brevity, using multiple mechanisms including advertisements in public places (e.g., libraries, grocery stores), pharmacies, health clinics, Internet-based classifieds (e.g., Kijiji.ca), and word-of-mouth. We were conscious of potential difficulties in recruiting participants and used existing literature regarding mechanisms to improve recruitment of potentially vulnerable or marginalized groups [48–52]. Inclusion criteria were that PLEs had to be able to understand and speak English, have experience with community pharmacy services for mental illness and/or addictions, such as with obtaining prescription medications, and at the time of the interview or focus groups, be considered as community-dwelling (i.e., not a resident of an institution). Recruitment occurred during June and July of 2012. Interested PLEs were offered the option of interviews or focus groups. We acknowledged that there are inherent differences and potential benefits and limitations with using both methods of data collection. However, our rationale and approach was informed by others conducting qualitative research with PLEs [53]. Some participants prefer face-to-face or telephone interviews depending on their cognitive abilities and comfort, and for others this method resembles the clinical encounter and therefore can distort the information that is shared [53]. Participants were paid a one-time honorarium of $20.00 (cash given at the interview or focus group) for participation.

We established a stopping criterion for data saturation [54] of eight to ten participants with the inclusion of three to five additional participants with no new information occurring. In interpretive description, and as indicated in the work of Thorne [43], the concept of saturation can be “highly problematic” (p.98), depending on the question, if researchers presume to have obtained sufficient data to understand all that is relevant about a clinical phenomenon, and in the case of our study, the experiences of people with mental illness and addictions in the community pharmacy context. However, we attempted to place a boundary on the potential size of the sample that was sound in principle and from a methodological perspective.

### Data collection and analysis

A semi-structured interview guide was developed (Additional file 1) based on our knowledge of the literature, our clinical experiences, and our study goals. The guide was reviewed by the members of the research team, refined, and revised based on feedback. The guide was then pilot-tested with four senior pharmacy students, a medical student with a Masters degree and research background in mental illness and addictions care, and two community pharmacists. Background information regarding the project’s purpose and objectives was explained prior to the examination of the guide for understandability, relevance, clarity, and fitness for purpose.

All interviews with participants, collected during June through September of 2012, were digitally audio-recorded, and subsequently transcribed verbatim. Field notes were written after each encounter. All transcription data were stored, organized, and coded with the use of QSR NVivo 10 [55]. We (ALM, DMG) followed procedures outlined for thematic analysis [44, 45]. The analysis stages included [44, 45]: familiarizing ourselves with the data through reading the transcripts along with the audio several times to develop “a sense of the whole beyond the immediate initial impression”(p.143) [43]; generating initial codes inductively with open coding and “flagging” data elements we thought were meaningful as we analyzed transcripts [43]; searching for themes, reviewing themes, defining and naming themes through bringing coded data together and “interrogating” [43] relationships among codes and themes. In the initial analysis stages, QSR NVivo 10 tools including word frequency queries and dendograms (i.e., depicting cluster analysis with codes by word similarity) were used [55]. Initial coding was conducted independently at first and subsequently over the course of ten, one to two-hour meetings, codes and theme development were refined, debated, interrogated, and finalized. To reduce bias and enhance confirmability, we used approaches such as analytic memo writing and engaging in discussions about our own subjectivity during data analysis as part of our audit trail. We also included all members of the team to review and critique the interpretation of the findings.

## Results

We collected approximately nine hours of audio data from 18 individuals using two focus groups of eight and four participants, respectively, and six individual interviews. The second focus group was scheduled for eight participants, but four did not attend. One person had an unexpected scheduling conflict and the other three could not be reached in follow-up.

Fourteen participants were female and the average age of participants was 41 years (range 24 to 57 years). All participants reported living with mental illness and some also indicated that they cared for others with mental illness and addictions either through employment or personal connections. We did not ask participants to specifically disclose their mental illness diagnosis or their medications. However, three people self-reported having addictions with one person reporting tobacco as the substance of their addiction. Participants had some difficulty estimating their visit frequency to pharmacies for health care-related needs. This was in part due to the changing nature of their health status, which varied the frequency of visits, and because they often went to pharmacies for other reasons (e.g., food items, personal hygiene products). With these considerations, estimates of visits to the pharmacy for prescriptions for mental health needs ranged from twice weekly to every three months. Two people (one from each focus group) reported not always using the same pharmacy for prescription-related needs. Three PLEs in the focus groups reported being uncertain of whether they were satisfied with their pharmacists’ services and two interview participants reported dissatisfaction with pharmacy team members as a reason for switching pharmacies, which resulted in better satisfaction at the new pharmacy.

### Themes: Expectations, decision-making, and supports

**Fig. 1:**
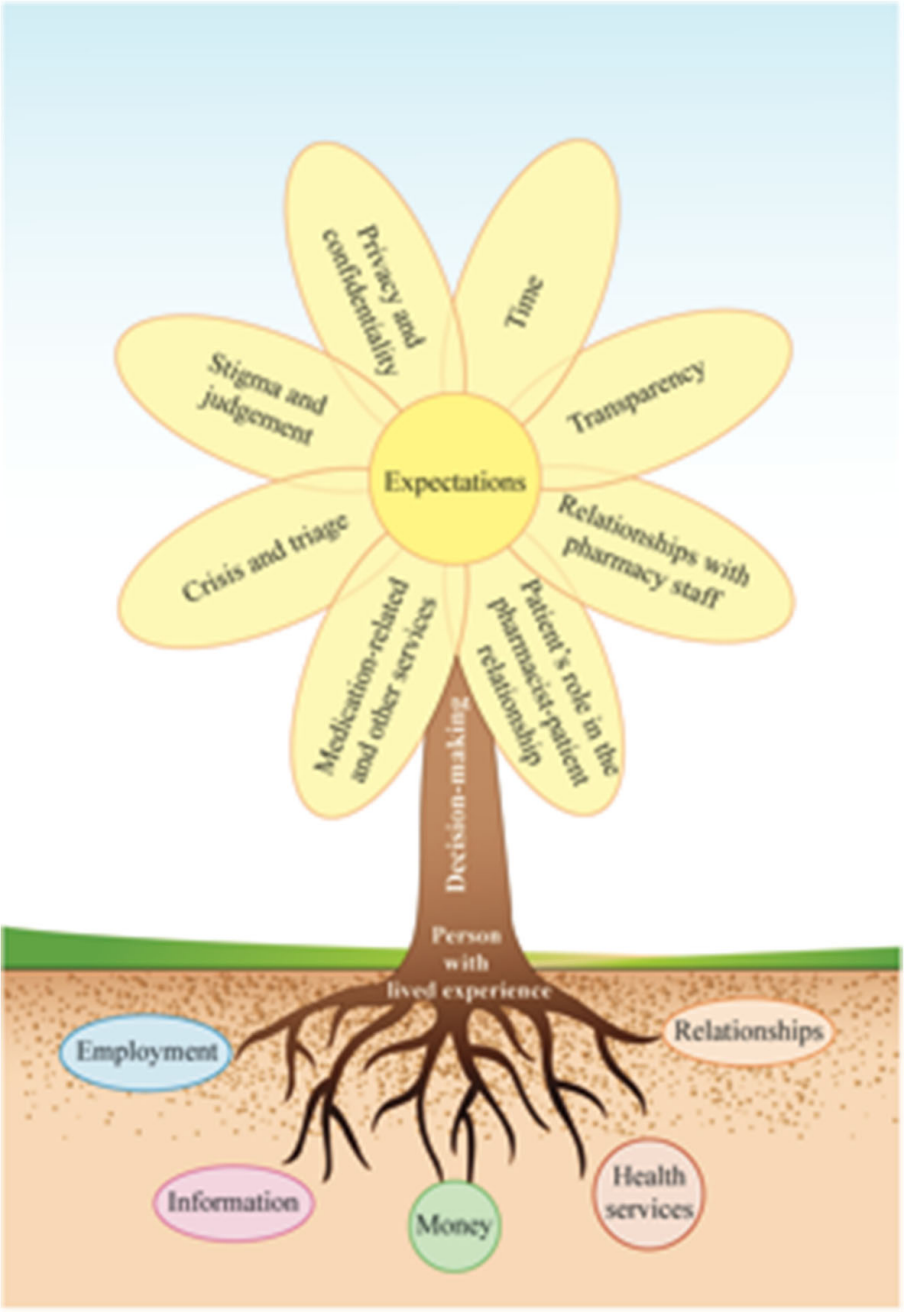
Representation of the experiences of people living with mental illness and addictions with community pharmacy services

We represented the product of our interpretive description of PLEs experiences with community pharmacy services in a metaphorical representation of the data, and within this, a modified Venn diagram [56] (Fig. 1). There are central elements to the experience of PLEs in accessing and receiving community pharmacy services, which include expectations, decision-making, and supports. Eight subthemes are linked among these overarching umbrella themes.

As depicted in Fig. 1, decision-making by PLEs in the pharmacy context was influenced by eight subthemes that centered on expectations. At the base of the experience were the supports (relationships, money, employment, health services, information), depicted as the fertile (or infertile) ground. These symbolized many required ingredients for health and wellness. These supports were critical to PLEs’ capabilities and opportunities with engaging in decision-making. The length of the stem is used to represent the amount of distancing between community pharmacies and the other supports for patients. A short, strong stem represents a well-integrated and connected community pharmacy and a long stem indicates a more isolated pharmacy that is disconnected from the community’s services and needs. The Venn diagram configured as the plant’s flower demonstrates the linkages among expectations and subthemes. The experiences of participants within each subtheme often overlapped and fed directly into reconstructing the PLEs’ expectations of pharmacy services and subsequently influenced their decision-making.

The majority of PLEs in our study expressed high expectations of pharmacists’ services compared to what they typically received, with several participants, but not all, being aware of or having experienced the full range of pharmacists’ capabilities. Participants’ expectations were in keeping with the pharmacists’ standards and scope of practice, ranging from those well within minimum standards of practice (e.g., technical aspects of dispensing) and those that would be considered more in keeping with clinically-oriented, patient-centred standards of practice (e.g., supporting patients with research literature and its interpretation for decision-making). Because of this, the decision-making process around medications and health-related concerns for PLEs was not always ideally supported. Participants also sought out different pharmacies when their expectations were not met, despite resulting inconveniences (e.g., travel) for some PLEs. Other participants accepted that their expectations would not be met based on rationalizations about the restrictions on pharmacists in practice (e.g., other demands on the pharmacist’s time).

### Subthemes

#### Relationships with pharmacy staff

Relationships that were built with pharmacists were important in the PLEs’ experiences and were viewed as an essential support in making decisions. For some PLEs, the linkages with pharmacists were critical in their mental health care as some other professionals were inaccessible, including prescribers.

*“ (Focus group 2, respondent 9; FG2R9) … my psychiatrist is only in once a week or twice. … He only books to see me every 4 months. … when I go to see him, he refills for the next year. … last spring, he gave me enough refills to get me through to next March. FG2R11: Wow! FG2R9: … I was only in there less than 5 minutes. It’s kind of ridiculous that he can … that he’s giving me refills until, like, the next year. It’s like it just seems a little absurd. … I don’t have a problem with it really but it’s kind of not really appropriate. And I find I rely on the pharmacy more than I rely on my own psychiatrist because he’s not there. … as soon as my psychiatrist does anything different, I hear from the pharmacy. Directly after, everything goes to the pharmacy. So as soon as something goes through to them, they’re always calling me and making sure that, you know, I know all the new changes.”*

Another PLE discussed her experience with and preference for working with a pharmacist with whom she had developed a good relationship. She spoke of her willingness to engage and share with this pharmacist, whom she trusted and respected, in contrast to the other pharmacists at the same location:

*“There’s one lady that is wonderful. She is. I could talk to her about anything. And she has gone to the end of the earth to get me help. Like at Christmastime, my drug plan for some reason just decided not to work, and I needed it [medication]… badly over the holidays. And she went to bat. She got everything ironed out like two days before Christmas. And she really, really, really went to bat for me. … But she’s one of maybe 6 people that work there. And I don’t have that same degree of comfort. So when she’s not working, I basically don’t even really talk about the medication with them. I just pick up my prescription and go on my way.” (Focus group 1, respondent 1; FG1R1)*

Another focus group participant described going to two pharmacies, one for food items, out of convenience, and one for her prescriptions, based on her long-standing relationship with the pharmacist:

*“I actually go to two pharmacies. I go to [pharmacy X] for like my groceries and I have a … [pharmacy Y], I know the man who runs the one out in [location]. … He’s really nice. I go there for my prescriptions.”(FG1R5)*

One PLE described using the pharmacy and pharmacy staff as an important part of her primary care health care because they knew her and her medications:

*“I mean that’s where I go if my knees hurt or I have a cough or anything like that. I go to the pharmacy. … You see your doctor when you can or when the pharmacist says you need to see your doctor or whatever. But I think often it’s a first point of contact … ” (Interview Respondent 1; IR1)*

Another PLE demonstrated through her example that strained relationships can occur with some, but not all members of the pharmacy team, after she had an intense argument with technicians regarding her medication insurance not working while processing a prescription:

*“I’ll be hesitant to fill a prescription after the run-in with the last two technicians. But when I see my regular pharmacist, I’m okay.” (FG2R12)*

For one PLE, several difficulties occurred in the relationship with her pharmacist but she felt unable to confront him and did not perceive she had a mechanism for providing the feedback. This significantly limited here relationship with the pharmacist:

*“Well, in particular, the pharmacist that I go to is very closed. … as far as their body language. So it’s not very easy to go to the person and speak to the person and talk to them.” (IR2)*

#### Patient’s role in the pharmacist-patient relationship

Some PLEs saw their own role in the relationship with their pharmacists as quite limited or had never considered it. Those that were able to discuss their role included descriptions of honesty or lack of disclosure, and most discussions related to medication supply. The patient’s role in the relationship depended on the quality of the relationship with the pharmacist.

In one focus group, participants discussed the need for honesty and communication in order to receive help:

*“FG2R3: You have to be honest with them. If they don’t know what’s going on, they can’t help you. Just like with your family doctor, if you don’t let them know, they can’t do anything. … FG2R2: … Communication is the key. Honesty is within that. Yes. So you have to be an advocate for yourself. FG2R1: That’s for sure. FG2R2: … Because nobody else can or would. FG2R: Yes. [group agreement] FG2R5: I guess this falls into the same – don’t die of embarrassment. … talk to somebody if there’s a medical issue, no matter how embarrassing it might be going on. Maybe there’s something the pharmacist could do to help. But if they don’t know, they can’t help you. You’ll still suffer with it.”*

One PLE with self-harming events associated with medications discussed disclosing the information openly after switching pharmacies and experiencing better relationships:

*“… I feel like I should be honest with what happens … I think the pharmacist before [at other pharmacy] was … I would say something other than that I overdosed. I’d say something like, “Oh, I lost my meds,” or something like that. And it was just in response to not wanting them to judge me … obviously they probably knew anyways. … they’re genuine with me and so I’m straightforward with them [new pharmacists]. … I don’t like having to like feel that I have to hide what’s going on. … I mean the fact that they’re willing to be … straight … That they’re comfortable with it is a big relief. … it would just feel silly to not be honest.” (IR6)*

#### Crisis and triage

Some participants discussed crisis and suicide assessment as a role for pharmacists. Situations were discussed in which patients had used medications in suicide attempts. In one situation, the PLE discussed that she was uncertain as to whether the pharmacists knew she had used medications as the means to attempt suicide despite having a significant hospital stay and being dispensed small quantities of medications following the attempt. Despite some trepidation regarding feasibility, she supported pharmacists’ training in suicide prevention:

*“… I have a really good relationship with him [doctor]. And I think that it’s important to have that type of relationship with your pharmacist, that they know you. … and they could say, “Is everything alright? Is there something you want to talk about?”… There’s a course before the ASIST* ^*^ *course. … SAFE-T* ^*†*^ *, that’s what it is! That’s the first one and then the ASIST is the second. … SAFE … to kind of recognize what’s happening and to get help. … So you know, to call [crisis service] or to call their psychologist or psychiatrist or something and make that connection and get them that help. … they [technicians] could even go to the pharmacist and say, you know, “[Participant] seems really off today,” or, “She didn’t come in and pick up [her prescription]” … Or anything, anything that they happened to notice about how you’re feeling or talking. So if they kind of have that awareness. In my mind, it’s better to be safe than sorry in terms of going to somebody and saying … or to ask your customer really how they’re doing. … And how much, as a pharmacist, do you want to take on? Do you want to become a mental healthcare worker at the same time? Although, I do believe that everybody in the healthcare sector and the public should have some suicide prevention training.” (IR1)*

^*^Applied Suicide Intervention Skills Training; ^†^ Suicide Assessment Five-Step Evaluation and Triage.

One participant nonchalantly described using the pharmacy as a resource for a range of things from culinary conveniences to a mental health crisis:

*“Usually it’s just for getting meds honestly. I mean it’s like a minute from my house so we get like staples there too, milk or whatever. I don’t know, sometimes I’ve had to go and ask like for their advice whether I should continue taking a med. Basically I sometimes overdose on my meds. And then it’s like okay, “well, when should I start taking my other meds?” (IR6)*

This participant also suggested roles in keeping with the pharmacist’s scope of practice with populations such as university students:

*“… that would be an awesome job of a pharmacist. … if someone is just on like a mild dose of antidepressants and they seem to be getting worse, it’s like they [pharmacists] should be able to point them in the direction of resources so that you don’t have a major episode. Because once you have an episode, it’s much more likely that you’ll have another episode.”*

#### Privacy and confidentiality

Privacy and confidentiality was an expectation by PLEs but several examples were shared in which this had not been afforded and this was a barrier to discussing medication-related issues. All participants discussed ideas regarding the use of private counseling rooms, albeit some participants were skeptical due to time restraints. Telephone conversations with pharmacists were frequently offered by participants as solutions to overcoming privacy in the pharmacy environment:

*“… having rooms to go in and discuss your drugs or having lots of access, you know, on the phone to talk about personal drug concerns… Instead of having to discuss it there with everybody standing there … is not so great. … If they could have that, it would be good. But it doesn’t sound reasonable unless they had a pharmacist just to do that. I think they would almost need that. … Interviewer (I): How do you feel about that [having medication discussions at the pharmacy counter]? IR5: A little intimidated. Other people standing there looking at you, listening. [laughs] … It is quite intimidating. I: In that situation, are you able to discuss what you need to discuss when you’re feeling that intimidation? IR5: Not always. But you know, there’s always the phone. You can always have the conversation on the phone if you need to. Right?” (IR5)*

#### Time

Some participants did not expect pharmacists to have time for discussing medications or other concerns. Issues with limited staffing were identified as one of several contributing factors impacting the pharmacists’ workloads:

*“I find there are not enough pharmacists. … there’s maybe one or there might be two if you’re lucky. But there should … be more of them because sometimes you can have … a couple of people with some complex problems in line, and you’re waiting, and waiting, and waiting, and waiting. It’s not particularly that pharmacist’s fault, it’s just that whoever is hiring them just didn’t hire enough pharmacists. It would be nice if there were like 2 or 3 of them.” (FG1R5)*

The manifestations of pharmacists under time pressure (e.g., running from task to task) formed the identity of pharmacists for some PLEs:

*“… the pharmacist at the place that I go is running. It’s the lady who’s running. You know, she seems to be running a lot. … [group laughter] … she seems really busy.” (FG1R1)*

Another PLE discussed the how “busy” the pharmacist was and that the use of the private counseling room given the time constraints would yield the pharmacist as inaccessible:

*“… They’re so busy now. If they had to go to a room, they would be in there forever.” (IR5)*

In discussing time constraints, some participants commented on the greater time spent with their pharmacist versus their doctor:

*“… they [pharmacists] spend more time with us. So they do know more about what’s going on and how we’re being affected by the medications … maybe they’re not specifically trained like a doctor but I’ve been in the doctor’s office for two minutes. … you often spend a bigger chunk of time talking to the pharmacist about what’s going on and relaying symptoms and different things that could occur, reactions with other medications. I think in some ways they [pharmacists] probably are a better advocate just because they do know you more. They see you more. … ” (FGR12)*

One PLE discussed being mindful of pharmacists’ and technicians’ time and purposefully went straight to the pharmacist with questions, regardless of the issue. This was perceived to be more efficient and in their experience, the pharmacists were accessible and made time for them:

*“I always ask for the pharmacist because why go through the middleman [technician]? … And there’s two main pharmacists where I go. And both will take time. I don’t need to make an appointment to talk to them, and they don’t seem to rush me. And my doctor doesn’t rush me either. And they’re connected in the same building, which is nice.” (FG1R2)*

#### Stigma and judgment

Several participants discussed stigma and judgment, with contrasting experiences and outcomes. Some people who experienced stigma were anticipatory of its recurrence and this altered how they interacted with pharmacists. Several participants chose to switch pharmacies or proactively interact with only selected members of the pharmacy team for services. This resulted in significant travel and other inconveniences for PLEs:

*“ … they didn’t mean for me to hear that but I could hear in the background, the pharmacist yelling, “… Just get her off the phone. She’s one of “those” [participant emphasis] people.” … they treated me like I was a bother. … I remember they short-changed my pills … and I could hear somebody talking in the background, “Oh, she’s mentally ill. Don’t take her too seriously.” [strong group reaction with gasps] … that was it! So I was just like, “you know what, I can get my drugs somewhere else”. And I switched from then on. … When he [another pharmacist] opened up a pharmacy, I’m like, “I’m coming to you because you’d never treat me like that!” … I know I could ask him … anything, and he doesn’t judge or he doesn’t get uncomfortable.” (FG1R5)*

A PLE expressed fear and anticipated judgment by pharmacists related to overdose with medications but was received with support:

*“… at the pharmacy I’m at now … I’d gone in before being like, “I overdosed and this is what is happening” sort of thing. So they know that. … they know my name and they ask me how I’m doing. You know, it makes a difference. … And I was really nervous the time after that going back in and being like, “oh my God, they’re going to judge me”… . But I mean no, they’re pretty good”. (IR6)*

#### Medication-related and other services

Participants inherently expected pharmacists to fulfill duties related to medications including provision of information as a part of routine services, but for some PLEs this was limited to initial medication education in which they were told information such as side effects and how to take the medication. Overall, there was limited information sharing in the pharmacist-PLE relationship and when it did occur, it appeared mainly focused on drug interactions and side effects. For many PLEs, activities such as dropping off prescriptions and picking up refill medications occurred primarily with technicians. Although there was a connection and rapport with a pharmacy team member for a “chat”, additional follow-up for sharing of clinical information with the pharmacists would have occurred only if the technician perceived a need based on the discussion with the PLE:

*“No, more time I think with the technician. … And there’s a couple at the drugstore that I go to. … they know me by my first name. And we chat and joke a bit, and that kind of thing. … And you know, bits about your personal life. … And I think personally that I might be more inclined to chat with them or tell them because I seem to have a more personal relationship with them. …” (IR1)*

Other PLEs viewed that pharmacists should be more engaged when patients are picking up refills:

*“I think they should ask you how you are doing because usually you see a pharmacist more often than your doctor. You’re going in at least once a month to pick up your pills. They should say, “How are you doing with that anyhow? Any changes?” Because they can be sort of your frontline. Like they might pick up something that’s wrong even before your doctor does, just because they see you so often. [group members agree]” (FG1R5)*

Most PLEs described a desire for more information to support their decisions regarding medication treatments. They were interested in pharmacists helping them access and interpret research relevant to their illness and its care. They indicated that they would like to see pharmacists proactively anticipate their information needs:

*“FG2R1: … anybody that could find me a study that says … the random whatever that double blind study is on that particular drug, and they gave 50 % a placebo and the other this, and this is the percentage of people that actually improved being on that drug. I would feel a whole lot more comfortable knowing that that particular scientific study is in place. And if the pharmacists can tell me that, that’s great. FG2R2: That’s what my doctor has done, like when I’ve been asking questions. Because there was a new medication, because I was newly diagnosed in the spring, and he pulled up case studies and then he gave me the reference. He read, you know, the highlights when I was in the doctor’s office but then he gave me the information and then I read it thoroughly myself at home on the computer too. … It can be results but unless they’re statistically significant, it doesn’t really count. … FG2R5: I like to have the evidence in hand. I don’t just take somebody’s word for it. … I want to see like an actual study. Because it’s my body and brain I’m putting on the line, I want to be sure that there was research done on it and what I’m taking isn’t dangerous, and there was actual quantitative results, something that could be measured. FG2R7: But can’t you seek that information on your own? FG2R5: You can but it’s hard because oftentimes when you look up a scientific paper, you have to pay for that paper. FG2R1: [interrupts and agrees] …. “the medical libraries” [participant emphasis]… FG2R5: You have to subscribe to the website or you have to pay for that paper. They’re not cheap. Sometimes they can be $25, $35 for that paper. FG2R1: If you can even get [participant emphasis] access. FG2R5: But sometimes pharmacists do have access. R: Yes [group agreement]. FG2R2: And they have more specific information on-hand so they can help you leaf through that. So then you can go in and select what you want to read. [long pause] Again, they have a wealth of information, and they need to be able to share it with you, particularly when you ask, and you shouldn’t even have to always ask, they should know sometimes to give you the information when you don’t know necessarily the question to ask. Because some people don’t or won’t.”*

Other PLEs similarly discussed using multiple sources such as health care professionals, including telephone conversations with pharmacists, and the Internet:

*“IR3: I use the Internet. I talk to the doctors, talk to the pharmacists. That’s the main sources. I: Do you have any favourite Internet sites? IR3: NIH, Mayo Clinic. … Like I say, I think that we definitely see pharmacists as a source of information. … how many people make use of that, I don't know. I would if I didn’t have my family doctor. I do go and ask questions and call and harass them [pharmacists]. Maybe that’s why they know my first name. [laughs]” (IR3)*

#### Transparency

Transparency issues manifested from two key areas in the context of pharmacy practice for PLEs: 1) issues with respect to the communication and clarity in medication procurement, preparation, record keeping, and payment processes; and 2) conflicts of interest with products or medications (e.g., linkages with pharmaceutical industry).

A lack of transparency regarding the business implications for a pharmacy procuring a relatively expensive medication left one participants feeling frustrated and stigmatized:

*“I had to get actually my psychiatrist to speak to her [pharmacist] about that because she was sort of chastising me because I was on this expensive drug. And indirectly, like non-verbally, like, “Oh, you know, that’s really expensive, you know. Like you’ve got to call and tell us. You know, that’s a special order.” And she kept saying how expensive it was. You know, implying that I shouldn’t be taking it. … And also one of the people that worked at the service desk [in the pharmacy] when I was getting a purchase order for an over-the-counter [medication] that had to go through the drug store, he sort of had the same opinion. … they know you’re on social assistance if you get a purchase order because they’re familiar with the process. And so it’s kind of like you’re less of a person if you’re on social assistance even though you’re working part-time and maybe hopefully in the future, you’re going to be working more and off the system, you know. … So they put you in a category … a lot of people … think people on social assistance sit home and drink beer all day. You know, a lot of people think that.” (IR5)*

Questions and lack of clarity around dispensing procedures were demonstrated through a discussion on a commonly occurring phenomenon in pharmacy practice in which generic manufacturers are changed from one prescription to the next depending on which brand is currently available at the pharmacy. Participants had identified that they noticed the changes in brands, which was a source of confusion regarding why brands were changing and without proper communication from the pharmacy to the PLE:

*“FG2R4: It will be like APO-sertraline and the next one will be… FG2R8: Mylan or whatever. FG2R4: Mylan. … Yes, I notice on the bottle, there’s always… It’s a generic. FG2R5: It’s just depending on who made that. Apotex is one manufacturer … the name before the dash is who makes it. FG2R8: Yes. I just figure like if you’ve been on the same one for a long time, and they use different manufacturers sometimes, they might want to check with people more often and see if they’re still doing okay when they have different manufacturers. FG2R5: Yes. Because they’re supposed to be the same but they’re not at times. FG2R8: Yes, because I’ve noticed I’ve felt different sometimes … or different side effects and things like that.”*

Transparency also became an issue when exploring roles for pharmacists in doing education sessions for the public. Participants discussed potential concerns regarding conflict of interest vis-à-vis the pharmacists’ roles in dispensing medications:

*“FG1R11: … if education sessions are like run by like pharmacists then it kind of just raises a red flag because everyone is just so wary of … the pharmaceutical industry with…. like all the money involved in pushing drugs. Like I think that maybe it should be run by someone who doesn’t have so much like financial interest in drugs, I guess. FG1R10: Yeah … or maybe pharmacists with some other types of, you know, psycho-social interventions of some kind could combine and talk together. And definitely not a drug rep. [group laughter]”*

## Discussion

Our interpretive description study is one of a few qualitative explorations of people with mental illnesses and addictions and their experiences with community pharmacy services. As such, it provides useful information that will inform intervention and program development. The flower, as our visual representation of the interpretive description for people with lived experience of mental illness, can be used when developing, implementing, and evaluating service development, making policy changes, and developing education and training opportunities for the pharmacy practice context. Importantly, our findings suggest that there is recognition and potential support for pharmacists’ roles in mental health care by the public to be “points of first contact” with the health system, more involved with facilitating informed decision-making, and equipped to participate in suicide risk assessment and mitigation.

Participants’ expectations of services were often higher than what was offered, which would be described as minimum standards of practice in many examples shared. Participants accepted, adjusted, and lowered expectations given their assumptions, experiences, and observations of pharmacists in the practice environment. Various barriers such as limited staffing, lack of privacy, and undeveloped/inadequate rapport, including issues with stigma, led many participants to rationalize not having expectations met in the pharmacy settings. Several people chose alternative mechanisms in how they interacted with pharmacists, such as using the telephone for talking with the pharmacist at a different time about their questions or concerns, and being selective in which pharmacist or pharmacy they chose. Interestingly, similar barriers (e.g., stigma, time) to the implementation of mental health services have been reported in studies examining perceptions of pharmacists and pharmacy staff [12, 20]. Based on our results, significant challenges in the pharmacist’s context of practice exist and impact pharmacist-PLE relationships. In order for pharmacists to ideally practice to their full scope in mental health care, as suggested by various organizations and policy documents [1], and even many participants in our study, adjustments in the structures and processes in the work environment are required.

Relationship quality between PLEs and pharmacists, and for some participants with technicians, was paramount. The quality of the relationship was imperative for information sharing and open communication around health and medications. There were several participants that disclosed information to pharmacists only out of necessity because of poor relationships due to stigma, inaccessibility, and conflicts. Other research has shown that patients may be more likely or willing to engage with pharmacy services depending on how well pharmacists listened to them in previous service encounters [57]. People are also more likely to build and sustain relationships with pharmacists when they are more satisfied about the quality of care they receive [58]. Positive relationship examples discussed by our participants were facilitated by various characteristics including the absence of stigma and judgment, sufficient time for discussion either in person or over the phone, adequate privacy, and perceived capabilities of the pharmacist. Lack of time and privacy have been reported by others as barriers to patient participation in education and consultations offered by pharmacists [59].

Participants in our study also desired to have more time and more access to be able to capitalize on the pharmacist’s knowledge for decision-making about treatments, including aspects such as whether a medication would be the correct one based on research evidence and other treatment options. This is particularly important given that medication-related information needs are not consistently addressed by health care professionals [60, 61] and that an overall movement in health care in recent decades is towards more patient-centred care and patient involvement in decision-making [62]. Patients can experience additional challenges in deciphering the quality and relevance of information among extensive quantities and types of health information that currently exist through various means (e.g., Internet). At times, patients may also lack awareness about what needs to be asked in clinical encounters, which was identified in this study and has been previously identified in youth with mental illness [63].

Participant PLEs shared information about their information resource use including health databases (e.g., PubMed), medical libraries, Internet sites, journal articles, and other resources. They discussed knowledge of evidence-based practice concepts and expressed desires to have help with accessing, interpreting, and applying this information in their decision-making. Previous research demonstrates that various issues with health literacy and numeracy can impact patients in understanding and applying evidence-based and other information in health care [64–67]. Satisfying PLEs’ information needs and facilitating decision support with the help of pharmacists did not, and was unlikely, to occur for many PLEs in our study given the nature of discussions around lack of staffing, time, and privacy.

The PLEs in our study also expressed an interest in having non-pharmacological alternatives (e.g., cognitive behavioural therapy) as treatment options and they saw pharmacists fulfilling roles in recommending other resources (e.g., community groups) outside of medications. Youth taking psychotropics have previously reported a desire for alternatives beyond medications and disclosed using a variety of interventions [68]. Other literature has demonstrated that pharmacists who understand these preferences and the patients’ perspectives on the meaning of medication may be better at patient-centred engagement and service provision [69].

For some patients, questioning medications, including asking whether the medication is necessary and exploring non-pharmacological therapies, may be a means of regaining a sense of control in their life after illness and medications create a shift in the person’s identity [68, 69]. For some PLEs, the act of taking psychotropics is viewed as an essential component of care but simultaneously stigmatizing [68–70]. The experience of stigma and judgment occurred and was feared by many of our participants leading to unnecessary emotional strain and alterations in accessing pharmacists’ services. Several PLEs took decisive action as a result of the experience of stigma by pharmacy staff and switched to another pharmacy. In these examples, stigma created emotional distress and frustration and led to challenges in finding a new pharmacy and establishing rapport and trust with another pharmacist. In other research of community pharmacy-based mental illness and addictions care in Canada and elsewhere, issues with stigma, although still present [12, 13, 16, 19, 23, 24, 71–74], may be on the decline with improved motivations and increased capabilities of pharmacists in mental illness and addictions care through the use of various interventions (e.g., education, training, enablement, modeling, etc. [42]) [18, 20, 75, 76].

Finally, an important subtheme in our findings was the notion of crises and triage by pharmacists, especially around suicide, including by self-poisoning, which was discussed by some of our participants. The research to date on the topic of pharmacist assessment and mitigation of suicide is practically non-existent and urgently requires attention [77], especially given trends regarding suicide attempts and suicides, including the use of medications as a means for suicide [77–87]. Recent study findings by Finkelstein et al. [87] highlight important lessons for community pharmacists who may be involved in the provision of medications that may be used in some self-poisonings (both prescription and non-prescription medications). Based on the data reported [87], two in five people overall, and one in five teenagers, died by overdose, after an initial self-poisoning episode requiring hospital care. Many of these people likely visited a pharmacy to obtain a medication for the treatment of a psychiatric illness. Others whom may have had a psychiatric illness would have visited a pharmacy to obtain a medication for a concurrent medical disorder (e.g., respiratory or cardiac disease, cancer, etc.). Thus, it is highly probable that pharmacists encounter patients at risk of suicide by self-poisoning and therefore a more comprehensive approach is needed for education, training, and research related to pharmacists’ roles in suicide risk assessment and mitigation [77].

## Limitations

One interviewee, who passed the screening checklist as community-dwelling, lived in a group home [88]. We included this transcript in the analysis as the contributions made to themes were within the scope of our study, but we also recognized that this group of PLEs would benefit from their own study regarding their interactions with pharmacists given their unique situation. Based on the participants in our study, we are also not able to make claims from our sample regarding substantive findings that are for people specifically with addictions (e.g., opioids or those receiving opioid replacement treatment). Although findings and our themes (e.g., stigma) may resonate with available literature on those with addictions, it is important to acknowledge this boundary both within our data and findings.

Our convenience sampling and recruitment methods may have led to a group of participants with a particular bias or motivation for participating.

We cannot claim that our interpretation of the data from individuals, in a specific context and health system, is the only one that exists.

## Conclusions

There are central elements to the experience of people with lived experience of mental illness and addictions in accessing and receiving community pharmacy services, which include modified expectations, a desire for pharmacist support in treatment decision-making, and health and wellness supports. Eight subthemes were identified including: relationships with pharmacy staff; patient’s role in the pharmacist-patient relationship; crisis and triage; privacy and confidentiality; time; stigma and judgment; medication-related and other services; and transparency. Research in the area of pharmacists’ roles in crises and triage, and especially suicide, is urgently needed. Future interventions for pharmacists and pharmacy staff may include components (e.g., education and training) to improve knowledge, attitudes, and behaviours with respect stigma and suicide risk assessment and mitigation. People with lived experience of mental illness and addictions have higher expectations of service delivery compared to what is often offered but are willing to adjust expectations given constraints they observe in community pharmacy practice.

## Additional file

Additional file 1: Interview guide for people with lived experience of mental illness or their caregivers. (DOC 33 kb)

## Abbreviations

PLE: Person with lived experience

## References

[1] National Mental Health Commission (2014). The national review of mental health programmes and services.

[2] Rubio-Valera M, Chen TF, O’Reilly CL (2014). New roles for pharmacists in community mental health care: a narrative review. Int J Environ Res Public Health.

[3] Mental Health Commission of Canada (2012). Changing directions, changing lives: the mental health strategy for Canada.

[4] Kirby MJL, Keon WJ (2006). The standing senate committee on social affairs, science and technology. Out of the shadows at last: highlights and recommendations.

[5] Laurant M, Harmsen M, Wollersheim H, Grol R, Faber M, Sibbald B (2009). The impact of nonphysician clinicians: do they improve the quality and cost-effectiveness of health care services?. Med Care Res Rev.

[6] Wazaify M, Shields E, Hughes CM, McElnay JC (2005). Societal perspectives on over-the-counter (OTC) medicines. Fam Pract.

[7] Law MR, Heard D, Fisher J, Douillard J, Muzika G, Sketris IS (2013). The geographic accessibility of pharmacies in Nova Scotia. Can Pharm J (Ott).

[8] Lynas K (2012). Professionals you can trust: Pharmacists top the list again in Ipsos Reid survey. Can Pharm J (Ott).

[9] Eades CE, Ferguson JS, O’Carroll RE (2011). Public health in community pharmacy: a systematic review of pharmacist and consumer views. BMC Public Health.

[10] Finley PR, Crismon ML, Rush AJ (2003). Evaluating the impact of pharmacists in mental health: a systematic review. Pharmacotherapy.

[11] Scheerder G, De Coster I, Van Audenhove C (2008). Pharmacists’ role in depression care: a survey of attitudes, current practices, and barriers. Psychiatr Serv.

[12] Murphy AL, Szumilas M, Rowe D, Landry K, Martin-Misener RM, Kutcher S, Gardner DM (2014). Pharmacy students’ experience in community pharmacy mental health services provision. Can Pharm J (Ott).

[13] Black E, Murphy AL, Gardner DM (2009). Community pharmacist services for people with mental illnesses: preferences, satisfaction, and stigma. Psychiatr Serv.

[14] Gardner DM, Murphy AL, Woodman AK, Connelly S (2001). Community pharmacy services for antidepressant users. Int J Pharm Pract.

[15] Bell JS, Aaltonen SE, Airaksinen MS, Volmer D, Gharat MS, Muceniece R, Vitola A, Foulon V, Desplenter FA, Chen TF (2010). Determinants of mental health stigma among pharmacy students in Australia, Belgium, Estonia, Finland, India and Latvia. Int J Soc Psychiatry.

[16] O’Reilly CL, Bell JS, Chen TF (2010). Pharmacists’ beliefs about treatments and outcomes of mental disorders: a mental health literacy survey. Aust N Z J Psychiatry.

[17] Wheeler A, Crump K, Lee M, Li L, Patel A, Yang R, Zhao J, Jensen M (2012). Collaborative prescribing: A qualitative exploration of a role for pharmacists in mental health. Res Social Adm Pharm.

[18] Crump K, Boo G, Liew FS, Olivier T, So C, Sung JY, Wong CH, Shaw J, Wheeler A (2011). New Zealand community pharmacists’ views of their roles in meeting medicine-related needs for people with mental illness. Res Social Adm Pharm.

[19] Knox K, Fejzic J, Mey A, Fowler JL, Kelly F, McConnell D, Hattingh L, Wheeler AJ (2014). Mental health consumer and caregiver perceptions of stigma in Australian community pharmacies. Int J Soc Psychiatry.

[20] O’Reilly CL, Bell JS, Kelly PJ, Chen TF (2015). Exploring the relationship between mental health stigma, knowledge and provision of pharmacy services for consumers with schizophrenia. Res Social Adm Pharm.

[21] Phokeo V, Sproule B, Raman-Wilms L (2004). Community pharmacists’ attitudes toward and professional interactions with users of psychiatric medication. Psychiatr Serv.

[22] Rijcken CAW, van der Veur H, Knegtering H, de Jong-van den Berg LTW (2003). Schizophrenia care and the Dutch community pharmacy: the unmet needs. Int J Pharm Pract.

[23] Knox K, Kelly F, Mey A, Hattingh L, Fowler JL, Wheeler AJ (2015). Australian mental health consumers’ and carers’ experiences of community pharmacy service. Health Expect.

[24] Treloar C, Fraser S, Valentine K (2007). Valuing methadone takeaway doses: the contribution of service-user perspectives to policy and practice. Drugs: Education, prevention, and policy.

[25] Renberg T, Wichman Tornqvist K, Kalvemark Sporrong S, Kettis Lindblad A, Tully MP (2011). Pharmacy users’ expectations of pharmacy encounters: a Q-methodological study. Health Expect.

[26] McMillan SS, Kelly F, Sav A, King MA, Whitty JA, Wheeler AJ (2014). Australian community pharmacy services: a survey of what people with chronic conditions and their carers use versus what they consider important. BMJ Open.

[27] Whitty JA, Kendall E, Sav A, Kelly F, McMillan SS, King MA, Wheeler AJ (2015). Preferences for the delivery of community pharmacy services to help manage chronic conditions. Res Social Adm Pharm.

[28] Couchenour RL, Carson DS, Segal AR (2002). Patients’ views of pharmacists as providers of smoking cessation services. J Am Pharm Assoc (Wash).

[29] Brown S, Henderson E, Sullivan C (2014). The feasibility and acceptability of the provision of alcohol screening and brief advice in pharmacies for women accessing emergency contraception: an evaluation study. BMC Public Health.

[30] Dhital R, Whittlesea CM, Norman IJ, Milligan P (2010). Community pharmacy service users’ views and perceptions of alcohol screening and brief intervention. Drug Alcohol Rev.

[31] Krska J, Mackridge AJ (2014). Involving the public and other stakeholders in development and evaluation of a community pharmacy alcohol screening and brief advice service. Public Health.

[32] Saari KM, Lindeman SM, Viilo KM, Isohanni MK, Jarvelin MR, Lauren LH, Savolainen MJ, Koponen HJ (2005). A 4-fold risk of metabolic syndrome in patients with schizophrenia: the Northern Finland 1966 Birth Cohort study. J Clin Psychiatry.

[33] Starkes JM, Poulin CC, Kisely SR (2005). Unmet need for the treatment of depression in Atlantic Canada. Can J Psychiatry.

[34] Vasiliadis HM, Lesage A, Adair C, Boyer R (2005). Service use for mental health reasons: cross-provincial differences in rates, determinants, and equity of access. Can J Psychiatry.

[35] De Hert M, Peuskens J, van Winkel R (2009). Mortality in patients with schizophrenia. Lancet.

[36] Kisely S, Quek LH, Pais J, Lalloo R, Johnson NW, Lawrence D (2011). Advanced dental disease in people with severe mental illness: systematic review and meta-analysis. Br J Psychiatry.

[37] Lawrence D, Kisely S, Pais J (2010). The epidemiology of excess mortality in people with mental illness. Can J Psychiatry.

[38] Kisely S, Campbell LA, Wang Y (2009). Treatment of ischaemic heart disease and stroke in individuals with psychosis under universal healthcare. Br J Psychiatry.

[39] Kisely S, Sadek J, MacKenzie A, Lawrence D, Campbell LA (2008). Excess cancer mortality in psychiatric patients. Can J Psychiatry.

[40] Kisely S, Smith M, Lawrence D, Cox M, Campbell LA, Maaten S (2007). Inequitable access for mentally ill patients to some medically necessary procedures. CMAJ.

[41] Kisely S, Simon G (2005). An international study of the effect of physical ill health on psychiatric recovery in primary care. Psychosom Med.

[42] Michie S, van Stralen MM, West R (2011). The behaviour change wheel: a new method for characterising and designing behaviour change interventions. Implement Sci.

[43] Thorne SE (2008). Interpretive description.

[44] Braun V, Clarke V (2006). Using thematic analysis in psychology. Qual Res Psychol.

[45] Vaismoradi M, Turunen H, Bondas T (2013). Content analysis and thematic analysis: implications for conducting a qualitative descriptive study. Nurs Health Sci.

[46] Guirguis LM, Chewning BA (2005). Role theory: literature review and implications for patient-pharmacist interactions. Res Social Adm Pharm.

[47] Cane J, O’Connor D, Michie S (2012). Validation of the theoretical domains framework for use in behaviour change and implementation research. Implement Sci.

[48] Furimsky I, Cheung AH, Dewa CS, Zipursky RB (2008). Strategies to enhance patient recruitment and retention in research involving patients with a first episode of mental illness. Contemp Clin Trials.

[49] Mapstone J, Elbourne D, Roberts I (2007). Strategies to improve recruitment to research studies. Cochrane Database Syst Rev.

[50] Robinson JM, Trochim WM (2007). An examination of community members’, researchers’ and health professionals’ perceptions of barriers to minority participation in medical research: an application of concept mapping. Ethn Health.

[51] Woodall A, Morgan C, Sloan C, Howard L (2010). Barriers to participation in mental health research: are there specific gender, ethnicity and age related barriers?. BMC Psychiatry.

[52] Ford JG, Howerton MW, Lai GY, Gary TL, Bolen S, Gibbons MC, Tilburt J, Baffi C, Tanpitukpongse TP, Wilson RF, Powe NR, Bass EB (2008). Barriers to recruiting underrepresented populations to cancer clinical trials: a systematic review. Cancer.

[53] Schulze B, Angermeyer MC (2003). Subjective experiences of stigma. A focus group study of schizophrenic patients, their relatives and mental health professionals. Soc Sci Med.

[54] Francis JJ, Johnston M, Robertson C, Glidewell L, Entwistle V, Eccles MP, Grimshaw JM (2010). What is an adequate sample size? Operationalising data saturation for theory-based interview studies. Psychol Health.

[55] QSR International Pty Ltd.: NVivo 10 data analysis software for Windows.

[56] Verdinelli S, Scagnoli NI (2013). Data display in qualitative research. International Journal of Qualitative Methods.

[57] Carter SR, Moles R, White L, Chen TF (2015). The impact of patients’ perceptions of the listening skills of the pharmacist on their willingness to re-use Home Medicines Reviews: a structural equation model. Res Social Adm Pharm.

[58] Sakharkar P, Bounthavong M, Hirsch JD, Morello CM, Chen TC, Law AV (2015). Development and validation of PSPSQ 2.0 measuring patient satisfaction with pharmacist services. Res Social Adm Pharm.

[59] Fleming ML, Ferries EA, Hatfield MD, Atreja N, Yucel A, Rane PP, Sharma M, Wang X (2015). Patients’ beliefs regarding counseling provided by community pharmacists: an application of the theory of planned behaviour. J Pharm Health Serv Res.

[60] Stevenson FA, Barry CA, Britten N, Barber N, Bradley CP (2000). Doctor-patient communication about drugs: the evidence for shared decision making. Soc Sci Med.

[61] Happell B, Manias E, Roper C (2004). Wanting to be heard: mental health consumers’ experiences of information about medication. Int J Ment Health Nurs.

[62] Butalid L, Verhaak PF, Boeije HR, Bensing JM (2012). Patients’ views on changes in doctor-patient communication between 1982 and 2001: a mixed-methods study. BMC Fam Pract.

[63] Murphy A, Gardner D, Kutcher S, Davidson S, Manion I (2010). Collaborating with youth to inform and develop tools for psychotropic decision making. J Can Acad Child Adolesc Psychiatry.

[64] Galesic M, Garcia-Retamero R (2010). Statistical numeracy for health: a cross-cultural comparison with probabilistic national samples. Arch Intern Med.

[65] Garcia-Retamero R, Galesic M (2009). Communicating treatment risk reduction to people with low numeracy skills: a cross-cultural comparison. Am J Public Health.

[66] Rothman RL, Montori VM, Cherrington A, Pignone MP (2008). Perspective: the role of numeracy in health care. J Health Commun.

[67] Davis TC, Wolf MS (2004). Health literacy: implications for family medicine. Fam Med.

[68] Murphy AL, Gardner DM, Kisely S, Cooke C, Kutcher SP, Hughes J (2015). A qualitative study of antipsychotic medication experiences in youth. J Can Acad Child Adolesc Psychiatry.

[69] Shoemaker SJ, Ramalho de Oliveira D (2008). Understanding the meaning of medications for patients: the medication experience. Pharm World Sci.

[70] Malpass A, Shaw A, Sharp D, Walter F, Feder G, Ridd M, Kessler D (2009). “Medication career” or “moral career”? The two sides of managing antidepressants: a meta-ethnography of patients’ experience of antidepressants. Soc Sci Med.

[71] Liekens S, Smits T, Laekeman G, Foulon V (2012). Pharmaceutical care for people with depression: Belgian pharmacists’ attitudes and perceived barriers. Int J Clin Pharm.

[72] Anstice S, Strike CJ, Brands B (2009). Supervised methadone consumption: client issues and stigma. Subst Use Misuse.

[73] Bell JS, Aaltonen SE, Bronstein E, Desplenter FA, Foulon V, Vitola A, Muceniece R, Gharat MS, Volmer D, Airaksinen MS, Chen TF (2008). Attitudes of pharmacy students toward people with mental disorders, a six country study. Pharm World Sci.

[74] Liekens S, Smits T, Laekeman G, Foulon V (2012). Factors determining social distance toward people with depression among community pharmacists. Eur Psychiatry.

[75] Cates ME, Burton AR, Woolley TW (2005). Attitudes of pharmacists toward mental illness and providing pharmaceutical care to the mentally ill. Ann Pharmacother.

[76] O’Reilly CL, Bell JS, Kelly PJ, Chen TF. Impact of mental health first aid training on pharmacy students’ knowledge, attitudes and self-reported behaviour: a controlled trial. Aust N Z J Psychiatry. 2011;45:549–57. 10.3109/00048674.2011.585454 21718124

[77] Murphy AL, Gardner DM, Chen TF, O’Reilly CL, Kutcher SP (2015). Community pharmacists and the assessment and management of suicide risk. Can Pharm J (Ott).

[78] Hamilton AA (2012). Detecting and dealing with suicidal patients in the pharmacy. Can Pharm J (Ott).

[79] Juurlink DN, Herrmann N, Szalai JP, Kopp A, Redelmeier DA (2004). Medical illness and the risk of suicide in the elderly. Arch Intern Med.

[80] Kodaka M, Inagaki M, Yamada M (2013). Factors associated with attitudes toward suicide: among Japanese pharmacists participating in the board certified psychiatric pharmacy specialist seminar. Crisis.

[81] Langlois S, Morrison P (2002). Suicide deaths and suicide attempts. Health Rep.

[82] Mann JJ, Apter A, Bertolote J, Beautrais A, Currier D, Haas A, Hegerl U, Lonnqvist J, Malone K, Marusic A, Mehlum L, Patton G, Phillips M, Rutz W, Rihmer Z, Schmidtke A, Shaffer D, Silverman M, Takahashi Y, Varnik A, Wasserman D, Yip P, Hendin H (2005). Suicide prevention strategies: a systematic review. JAMA.

[83] Matsumoto T (2013). Current situation of suicide in Japan, and what pharmacists contribute to suicide prevention. Yakugaku Zasshi.

[84] Mental Health Commission of Canada (2015). Suicide prevention.

[85] Statistics Canada. Suicides and suicide rate, by sex and by age group (males rate, 2014). http://www.statcan.gc.ca/tables-tableaux/sum-som/l01/cst01/hlth66e-eng.htm. Accessed 15 Mar 2015.

[86] Statistics Canada. Suicides and suicide rate, by sex and by age group (females rate, 2014). http://www.statcan.gc.ca/tables-tableaux/sum-som/l01/cst01/hlth66f-eng.htm. Accessed 15 Mar 2015

[87] Finkelstein Y, Macdonald EM, Hollands S, Sivilotti ML, Hutson JR, Mamdani MM, Koren G, Juurlink DN, Canadian Drug Safety and Effectiveness Research Network (CDSERN) (2015). Risk of suicide following deliberate self-poisoning. JAMA Psychiat.

[88] Nova Scotia Government. Licensed Homes for Special Care. http://novascotia.ca/coms/disabilities/HomesForSpecialCare.html. Accessed 12 Mar 2015.

