# Supplementary material for: From personal crisis care to convenience shopping: an interpretive description of the experiences of people with mental illness and addictions in community pharmacies
Source: BMC Health Serv Res. 2016 Oct 12;16:569. doi: 10.1186/s12913-016-1817-4 (PMC5059973; doi:10.1186/s12913-016-1817-4)
Supplement: Additional file 1: — Interview guide for people with lived experience of mental illness or their caregivers. (DOC 33 kb) [file 12913_2016_1817_MOESM1_ESM.doc]

# Additional file 1: Interview guide for people with lived experience of mental illness or their caregivers.

**Question 1. People go to the pharmacy for many different reasons. Tell me about the reasons that you would visit your community pharmacy?**

***Probing Questions:***

- - - Are the services you get primarily related to medications? What other things do you do at your pharmacy? What services do you get?
    - Give an example of something you discuss with your pharmacist.
    - What services (refer to list of services at the end for examples*) would you like to get at your community pharmacy?

**Question 2. Tell me about the experiences you have had with community pharmacy team members (pharmacists, technicians, students) when seeking help for mental health and/or addictions related concerns or medications.**

***Probing Questions:***

- - - Describe an example of an encounter you have had with a pharmacist, pharmacy technician, or pharmacy student.
    - Do you perceive there are differences among or between the pharmacy staff members depending on their title in how they serve you or what they provide?
    - Discuss differences, if any, between your encounters with pharmacy team members for physical versus mental health and/or addictions problems.

**Question 3. How would you describe your relationship with members of the community pharmacy team?**

***Probing Questions:***

- - - How comfortable do you feel asking questions?
    - Have you experienced stigma in the pharmacy setting? Tell us what happened.
    - How well does the pharmacist listen to and address your concerns? What about technicians? Students?
    - How would you describe your relationship with the pharmacists?
    - How would you describe your relationship with the pharmacy technicians?
    - How would you describe your relationship with the pharmacy students? How would you describe your relationship with the front store staff (e.g. cashiers)?

**Question 4. Can you describe your role in your relationship with the pharmacy team? What kinds of things do you need to do in order for the pharmacy team to provide you with the best service?**

***Probing Questions:***

- - - What do you feel are your responsibilities (e.g. fully disclosing information about medications and medication history)?

**Question 5. Tell me about what you would see as ideal services that a community pharmacy team could provide for you.**

***Probing Questions:***

- - - Provide us with one or more example(s) of something that you would value and use if available.
    - What should pharmacists and pharmacy team members do for you? What is their role in your health care?

*** List of potential services to be shown at focus group:**

- Medication bubble/blister packages
- Reminders (electronically, in person, via phone)
- Telephone call back services to see how I am doing
- Home delivery of prescriptions
- Health promotion services (e.g. smoking cessation, helping with problem drinking)
- Education sessions on health conditions and wellness
- Providing meeting space for community support groups
- Coming to my child’s school to discuss mental health concerns of youth
- Providing lists of referral services for mental health
- Helping me communicate my medication and/or health concerns with my doctor or other health care team members
- Helping to ensure that my medications are the right ones for me
